# Minimally-destructive atmospheric ionisation mass spectrometry authenticates authorship of historical manuscripts

**DOI:** 10.1038/s41598-018-28810-2

**Published:** 2018-07-26

**Authors:** James Newton, Gordon Ramage, Nikolaj Gadegaard, William Zachs, Simon Rogers, Michael P. Barrett, Gerard Carruthers, Karl Burgess

**Affiliations:** 1 Glasgow Polyomics, University of Glasgow, Glasgow, UK; 2 Wellcome Centre for Molecular Parasitology, Institute of Infection, Immunity and Inflammation, University of Glasgow, Glasgow, UK; 3 Dental School, University of Glasgow, Glasgow, UK; 4 School of Engineering, University of Glasgow, Glasgow, UK; 5 School of Literature, Language and Culture, University of Edinburgh, Edinburgh, UK; 6 School of Computing Science, University of Glasgow, Glasgow, UK; 7 School of Critical Studies, University of Glasgow, Glasgow, UK; 8 Institute of Infection, Immunity and Inflammation, University of Glasgow, Glasgow, UK

## Abstract

Authentic historic manuscripts fetch high sums, but establishing their authenticity is challenging, relies on a host of stylistic clues and requires expert knowledge. High resolution mass spectrometry has not, until now, been applied to guide the authentication of historic manuscripts. Robert Burns is a well-known Scottish poet, whose fame, and the eponymous ‘Burns Night’ are celebrated world-wide. Authenticity of his works is complicated by the ‘industrial’ production of fakes by Alexander Smith in the 1890s, many of which were of good quality and capable of fooling experts. This study represents the first analysis of the inks and paper used in Burns poetry, in a minimally destructive manner that could find application in many areas. Applying direct infusion mass spectrometry to a panel of selected authenticated Burns and Smith manuscripts, we have produced a Support Vector Machine classifier that distinguishes Burns from Smith with a 0.77 AUC. Using contemporary recipes for inks, we were also able to match features of each to the inks used to produce some of Burns’ original manuscripts. We anticipate the method and classifier having broad application in authentication of manuscripts, and our analysis of contemporary inks to provide insights into the production of written works of art.

## Introduction

Traditional analytical methods for analysing ink involve destroying the sample through extraction procedures and then analysis via thin-layer chromatography (TLC) or liquid chromatography. More recently, several atmospheric ionisation mass spectrometry techniques have been investigated for the analysis of modern inks. These methods include desorption electrospray ionisation (DESI)^1^, direct analysis in real time (DART)^2^, laser desorption ionisation (LDI)^3^ and matrix-assisted laser desorption ionisation (MALDI)^4^, but no high-resolution mass spectrometry method has been applied to historical inks. We demonstrate here a method for the analysis of historic writing inks and paper using liquid extraction directly from the paper surface, analysed using direct infusion nanospray mass spectrometry. The advantages over the existing methods are such that no matrix is required and that sampling can be performed rapidly without needing to move the historic artefact, as it can be performed outside of the laboratory.

Historic documents can have great significance in many respects. They can, for example, give insight into the opinions of leading individuals whose influence on society is profound. In some cases they might be of legal importance, underpinning ownership of land or valued property. A global fascination with the works of great writers has made original handwritten documents items of considerable value too.

Scotland’s ‘national’ bard, Robert Burns (1759–96) is a world-renowned writer whose fame as a song-writer and a lover of women, as well as one of the first celebrities of the Romantic age, makes him a figure of enduring fascination. His first collection, Poems, Chiefly in the Scottish Dialect (1786), sells for anything between £40,000 and £80,000, when – as rarely happens – one comes on the market, one selling on 28th October 2010 at Sotheby’s for £73,250^5^. A single manuscript of a letter or poem by Burns might fetch £6,000 to £90,000 (the Mitchell Library, Glasgow, paid a seven figure sum in the past decade to acquire a manuscript (one of several) of ‘Auld Lang Syne’). The latest estimate of Burns’s value to the Scottish economy derived over a decade ago reached a figure of £157,000,000 per annum^6^. This figure would undoubtedly now be much higher, and did not take account of the international market for rare copies, manuscripts and memorabilia of Burns. The finer auctioneers of London, New York and elsewhere frequently feature such items in their sales. Burns has had several forgers, the most notorious of whom, Alexander Howland (‘Antique’) Smith served a prison spell for his efforts in the 1890s^7^. Many examples of his handiwork remain at large and, for instance, the New York Public Library, has a large collection of Smith’s ‘Burns’ manuscripts, now valuable in their own right but originally acquired in the late nineteenth century as genuine. Famous (or any) handwriting can be difficult to authenticate, as the Antique Smith and other cases demonstrate^8^. A skilled draftsman such as Smith can fool even experts for a time, indeed, the Earl of Rosebery (UK Prime Minister 1894–1895) bought Smith manuscripts sold as authentic Burns.

Deeper investigation, however, can sometimes identify inconsistences (as happened in the Smith case, where he failed to spot that Burns’s hand went through four identifiably different phases through his career). The colour of ink, authentic watermarked and aged paper (and especially lines in the paper) are all telling of an authenticity (along with other things) that require considerable expertise to fake^9^. However, these methods for assessing authenticity require considerable expertise, and the existence of a chemical model to distinguish real from faked manuscripts could have a considerable effect on the field of authenticity with important economic impacts.

When analysing unique and precious manuscripts it is important to minimise damage to them. X-ray fluorescence spectrometry (XRF), which measures the quantities of certain elements and impurities and gives a chemical fingerprint has been used in the identification of iron gall inks^10^, pigments and inks on oil paintings^11^ and coloured pencils^12^. XRF, however, is confined to analysing metallic elements in the substrate. Another minimally or non-destructive method, Raman microscopy, has been used to study the degradation of iron gall ink on manuscripts^13,14^, but the chemical information it can give is very restricted although it is more powerful in distinguishing coloured pigments in manuscripts^15^.

In order to obtain greater chemical information there has been more focus recently in using non-destructive atmospheric ionisation mass spectrometry techniques to interrogate ink and paper surfaces. In forensic science desorption electrospray ionisation (DESI) has been used to characterise a range of modern blue and black ballpoint, gel and rollerball inks^16,17^. Direct analysis in Real Time (DART) is another minimally-destructive technique which has been established as a document authenticity technique^18^. One of the major advantages DESI has over DART is the imaging capability for the analysis of documents which may have been altered at different time points^19^. As of yet however the analysis of historic black writing inks such as iron gall and ivory black has not been documented for analysis by minimally-destructive atmospheric ionisation mass spectrometry techniques.

**Figure 1:**
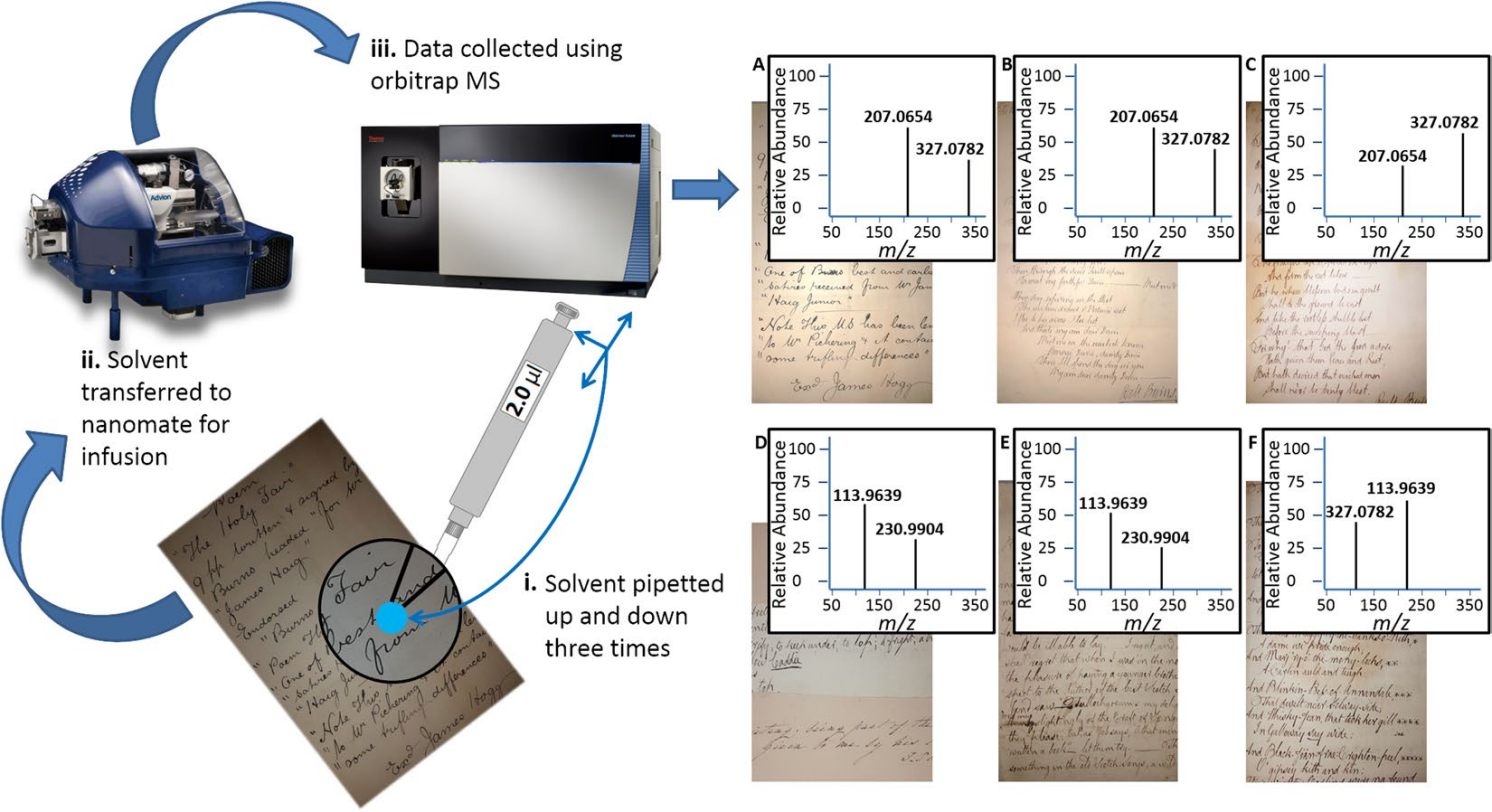
Schematic of the extraction and analytical process with direct Infusion data for authentic manuscripts. All samples for Antique Smith (**A**–**C**) have an ions at *m/z* 327.0782 and 344.1049; (**A**). The Holy fair in the hand of R. Burns, also signed by J. Hogg (**B**). Dainty dive poem in the hand of R. Burns and (**C**). The first psalm in the hand of R. Burns. All samples for Robert Burns have ions at *m/z* 113.9639 and 272.0655 (**D**–**F**); (**D**). A note written by R. Burns, (**E**). A letter written by R. Burns and (**F**). The five carlins poem.

Here we use direct infusion nanospray mass spectrometry for the first time to investigate the poetry of Robert Burns and distinguish it from manuscripts written by a skilled, contemporary forger. Authentic Robert Burns documents “A letter written to ‘Rev and Venerable Sir’” (MSS11), “A note” (MSS10) and “The Five Carlins” poem (MSS14) were sampled minimally-destructively from both ink and paper and subjected to analysis using ultra-high resolution mass spectrometry. These were compared to seven different forgeries produced by Alexander Smith. Baseline authenticity for both Smith and Burns was obtained using the gold standard methods of provenance and transmission of ownership, as well as expert inspection of the documents. Figure 1 shows a schematic of the technique used for the analysis with two of the most significant features distinguishing Smith and Burns highlighted.

**Figure 2:**
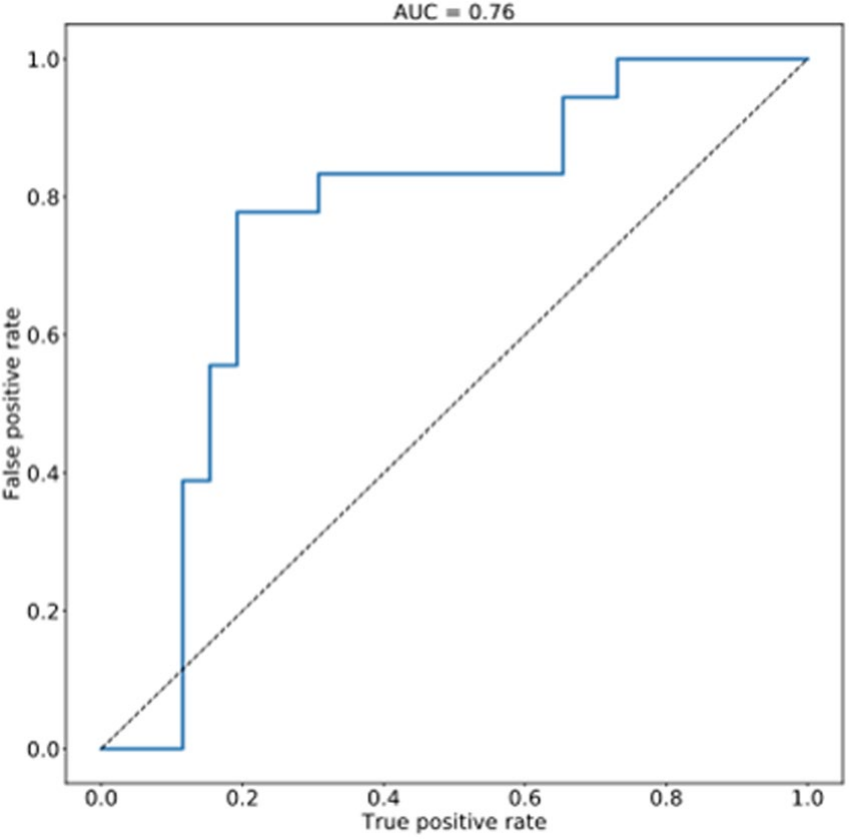
ROC curve demonstrating the sensitivity, specificity and false positive rate for a classifier based on mass spectrometric discrimination of Burns and Smith. To generate the model, a hard margin Support Vector Machine classifier was used with an rbf kernel function. Signals from both ink and paper were combined in this instance.

To assess the ability of untargeted spectral analysis to distinguish between real and forged documents, a classifier was produced. It is able to distinguish between real Burns and the Alexander Smith forgeries with an AUC of 0.77 (see Fig. 2). Peaks were selected as features if they were present in at least three samples and in none of the solvent blanks, resulting in 496 peak features. Classification performance was evaluated using a cross-validation procedure in which samples from a single document were held out as test data (to replicate the use case of training on documents of known provenance and testing on a new document) and the remaining documents were used for training. A hard margin Support Vector Machine classifier was used with an RBF kernel function^20^. Both paper and ink samples were combined in the classifier.

**Figure 3:**
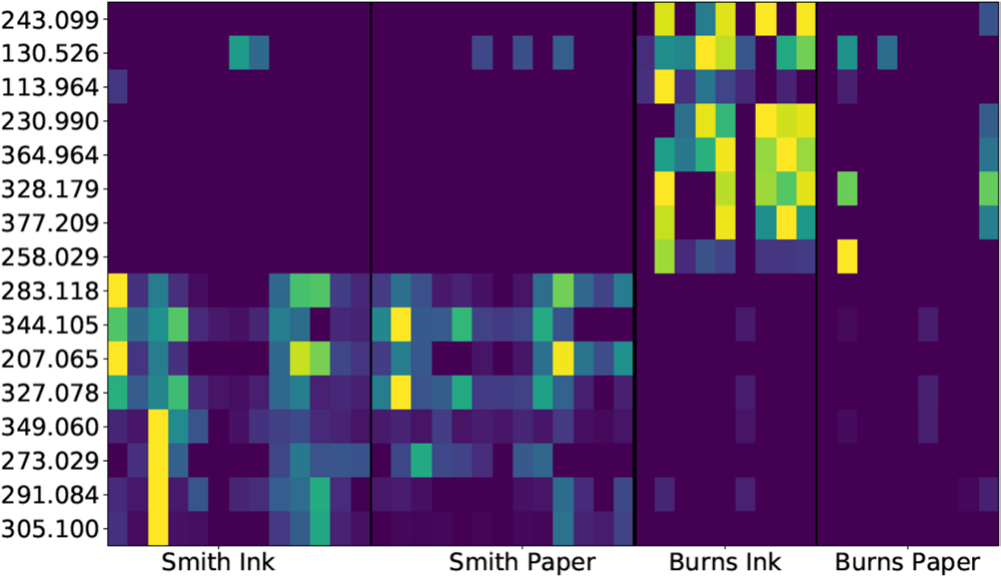
Heatmap of features found between Burns inks and paper and Smith inks and paper.

To further explore the discriminatory peaks, 94 significant differences (Q < 0.05) were found distinguishing Burns and Smith manuscripts, with 42 features not detectable in any solvent blank. After manual analysis of each significant peak not found in the solvent blanks, we selected eight from each author (see Fig. 3 and Table 1) that were high intensity, not isotopic peaks derived from other compounds and that were only detected in the respective author’s work. While many of the distinguishing features could not be annotated due to the minimal sample volume, predicted formulae were suggested for eight compounds, with annotations (obtained from ChemSpider) available for four of these. Two compounds are clearly organically-derived, probably from plant extracts (citropen and citrate), while the other compounds are potentially inorganically derived. Significant differences between the inks and paper were also detected in Burns, with a majority of features found in the paper also detected in the ink samples. There was little significant difference between ink and paper samples extracted from the Smith manuscript, likely due to his habit of tea- or tobacco-staining paper to artificially age it, producing strong features from the staining that overwhelmed signatures from the ink.

**Table 1:** List of diagnosing peaks, their predicted formulae, and, where available, a suggested compound name.

A. Smith	Annotation	Predicted Formula	R. Burns	Annotation	Predicted Formula
207.0654	Citropen/Scoparone - natural plant metabolite	C_11_H_10_O_4_	113.9639		???
273.0289		???	130.5259		???
283.1178	Bis(2-methoxyethyl) phthalate	C_14_H_18_O_6_	230.9904	Citric acid (Potassium adduct)	C_6_H_8_O_7_ + K^+^
291.0842		???	243.0993		C_11_H_10_N_6_O
305.0996		???	258.0286		???
327.0782	Triphenyl phosphate	C_18_H_15_O_4_P	328.1794		C_14_H_25_N_5_O_2_S
344.1049		???	364.9642		???
349.0602		C_14_H_12_N_4_O_5_S	377.2091		C_21_H_24_N_6_O

**Figure 4:**
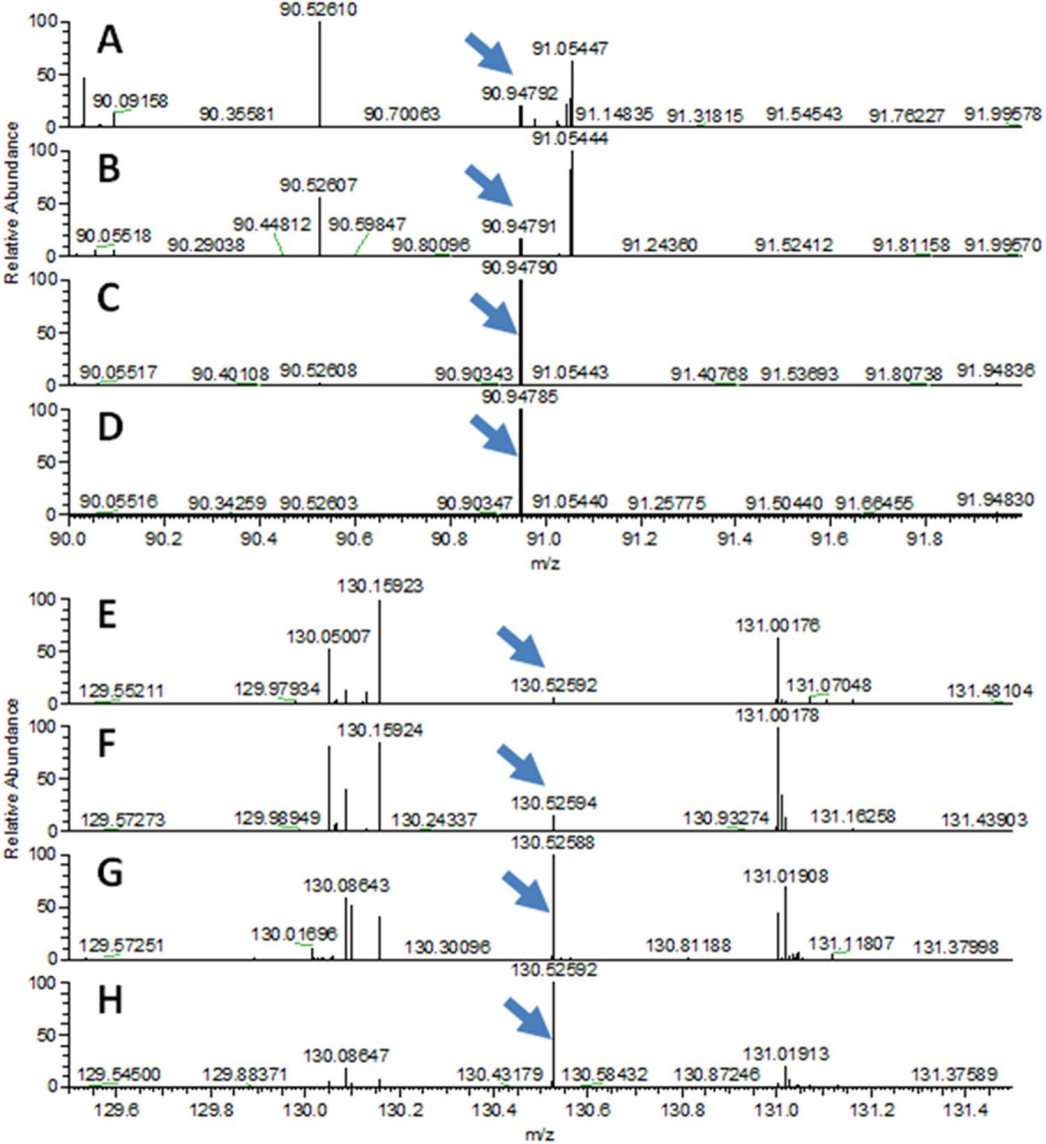
(**A**–**D**) Comparison of ink spectra to identifying peak for iron gall at *m/z* 90.9479 (**A**). A. Smith - The Holy Fair in the hand of R. Burns – also signed by J. Hogg. (**B**) A letter written by R. Burns. (**C**) Ink spectrum for simple ivory black. (**D**) Ink Spectrum for ivory black made with treacle. (**E**–**H**) Comparison of ink spectra to identifying peak for ivory black at *m/z* 130.5259 (**E**). A. Smith - Dainty dive poem 2 in the hand of R. Burns. (**F**) The Five Carlins poem written by R. Burns. (**G**) Ink spectrum for Blots iron gall. (**H**) Ink Spectrum for home-made iron gall.

After obtaining contemporary recipes for inks, especially iron gall and ivory black, we were able to match individual documents to ink signatures. In the The Holy Fair manuscript by A. Smith and in the letter written by R. Burns, we detect iron gall ink, and in the Dainty dive poem by A. Smith, we are able to detect the presence of ivory black, as shown in Fig. 4. Interestingly, in the Five Carlins manuscript by R. Burns, we detect both features, demonstrating that Burns, as was common at the time, mixed inks to obtain a desired lustre and consistency in his writing.

We anticipate applying the analysis in a tiered manner, such that an area of the document may be analysed with no effect on the written material, and if (as was commonly done by Smith) a forgery has been written on a document excised from an older, Burns-contemporary work, the ink may be used in a minimally destructive analysis to further clarify the authenticity.

Burns is a significant part of Scotland’s national heritage, and the provenance and authenticity of his manuscripts is a strength of collections both public and private. Manuscripts of dubious authenticity continue to appear at auctions, and a robust and quantifiable method that distinguishes the most prolific forger from authentic Burns is a significant step in this direction. Extreme caution is exercised over the discovery of any new potentially authentic manuscript: appearance is scrutinised and contents and ownership, if incomplete in record, usually can at least be credibly hypothesised. For approximately 80 per cent of the Burns corpus of manuscripts, it is possible to account for full provenance. However, 20% of manuscripts have less strong authenticity and a chemical classifier such as the one described in this paper will have a significant impact, not only within the field of Burns, but in literary authenticity in general.

To the best of our knowledge this is the first high resolution mass spectrometry analysis of contemporary inks and historical manuscripts. Signatures from the inks produced using historical recipes and original manuscripts from Robert Burns and ‘Antique’ Smith were compared in order to create a platform which questioned manuscripts can be authenticated. Techniques such as XRF, DART and DESI^15,16,18^ must be performed in a laboratory environment as a mass spectrometer is impracticable to move about and sources such as DESI can take time to fully optimise^21^. The simplicity however of the sample preparation method described herein means that the sampling can be easily performed at the site where the manuscripts are stored, which in turn could make it an ideal technique for auction houses to confirm authenticity.

## Methods

### Sample Analyte Extraction

Analytes from the ink on the manuscript surface were extracted by placing a pipette tip just above the surface, with 2 µl of solvent (methanol: water 60:40 + 0.08% formic acid), and pipetting up and down three times before transferring to the sample well for analysis as summarised in Fig. 1. Care was taken not to touch the sample surface. Extraction was performed in triplicate in different areas for each separate injection to collect enough sample and diluted to 8 µl using the same solvent.

### Mass Spectrometry

Direct infusion was performed using an Advion Triversa nanomate set up for chip based infusion on a Thermo Fusion Orbitrap. Spectra were collected for 1.5 minutes in positive mode over a scan range of *m/zm/z* 70–700. Ion transfer tube was set to 275 °C, RF lens 60% and maximum injection time of 50 ms. Fragmentation data was collected using quadrupole isolation at HCD collision energy of 60% and resolution 15000.

### Interpretation of Raw Data

Vendor raw files were converted to mzML and centroided using Proteowizard’s convert function (with the cwt centroiding algorithm). MzML files were processed in Python using PyOpenMS. In each file, (*m/z*, intensity) tuples were extracted using OpenMS’s mass trace detection algorithm (that finds *m/z* values that are consistently present across the injection period). Default parameter values were used for both the centroiding and mass trace detection. The resulting spectrum for each file was normalised to relative intensities by dividing by its total usable signal (total intensity), after which intensites were scaled by a factor of 1000. Peaks were aligned across the files using a greedy algorithm that sorted all peaks from all files by *m/z* and then moved along the *m/z* range collecting peaks into a peakset. A peakset was completed and the next peak placed in a new peakset when its *m/z* was more than 5 ppm away from the mean *m/z* value of peaks already in the peakset. If a file contributed more than one peak to a peakset, the most intense peak was retained. Differential expression analysis was computed across the experimental groups using a t-test on the log-transformed intensities (a small value (1e–5) was imputed into the missing values prior to logging). The resulting p-values were corrected using the Benjamini-Hochberg^22^ False Discovery Rate correction method.

### Classification Analysis

After aligning all peaks detected across the complete sample set, hard margin support vector machine (SVM) classifiers were built including all peaks that were present in at least three spectra and absent in all blank spectra. Performance was evaluated using a leave-one-out cross validation scheme, however in this case all technical replicates of a particular sample were held out together to ensure that technical replicates from the same sample were not simultaneously in both training and test sets. The SciKit-learn implementation of the SVM was used with an radial basis function (RBF) kernel and automatic kernel parameter tuning. A receiver operator characteristic (ROC) curve was produced by combining the predictions from each cross validation iteration.

### Ink recipes

Three of the inks were made using recipes either found online or in a book from the 18th century and one was bought ready made using an authentic recipe.

### Blots iron gall

Commercially available ink made using a medieval Palatino formula of 1540, this ink is an extremely acidic iron gall ink based on the following recipe:

“To make ink, take three ounces of galls, which should be small, solid, and wrinkled, and crush them coarsely. Steep them in half a flask of wine or, better still, of rain-water and allow them to soak in the sun for a day or two. Then take two ounces of copperas (Ferrous sulphate) or Roman nitre of a good, rich colour and finely crushed. Stir the galls with a stick of fig-wood and add them in, leaving the mixture in the sun for a further day or two. Now, stirring the mixture up again, add an ounce of gum-arabic, which should be clear, lustrous, and well-ground. Leave for a whole day. To make it nice and bright, add a few pieces of pomegranate peel and bring to the boil over a very slow fire; then strain it and keep it covered up in a glass or a lead container; and it will be perfect. “ Extract from: Osley, A. S. (1980). *Scribes and Sources*. London: Faber and Faber Limited. It is an oak gall ink recipe by Giovambattista Palatino (1515–1575, Italy).

### Homemade iron gall

A simple iron gall recipe was found on irongallink.org using recipe 2, taking about 4 hours to prepare, the recipe was adapted slightly as follows:

2 g of crushed oak galls and 150 ml of water was stirred and gently heated on a stirring hot plate for approximately 2 hours, ensuring that the solution does not boil or become dry and burn throughout the heating by adding further water as necessary. Once cooled this solution was then filtered and 1 g of Iron Sulfate was added to give a deep black colour. 35 ml of this solution was then diluted to 50 ml using a ready made gum arabic solution (Winsor & Newton) to give an ink of appropriate constitution.

### Simple ivory black

A very quick and simple ink to prepare, however requires stirring periodically whilst using as the ink will settle out to the bottom. 1 g of Ivory Black (Kremer Pigment GmbH) was ground with a pestle and mortar with 10 ml of gum arabic solution (Winsor & Newton), this was then diluted to 25 ml with water to give a solution of the correct consistency.

### Ivory black with treacle

This is perhaps one of the most involved inks and was transcribed from an Unknown Author (circa. 1800), titled Ink Recipes:

“Directions for making Blacking given to me by W Francis Linn Draper Holnorn, next door but one to the Great [Turnstile], says it’s the same Day of Martins. ¼ lb Ivory Black, ½table spoonful Dripping, 1Oz Oil Vitriol, ¼ lb treacle, 1 pint Stale Table Beer or then add vinegar & stir well up, then add Oil Vitriol & stir all well together.”

The recipe was adapted as follows: 10 g Ivory Black, 1 g dripping and 10 g black treacle was measured into a 250 ml beaker along with 50 ml Stale Beer and stirred well on a stirrer plate for half an hour, then 2 ml Sulphuric acid was added and stirred for a further 10 minutes. The solution was then left to stand and any fat deposits were skimmed from the top before storing.

### Data Availability Statement

The datasets generated during and/or analysed during the current study are available in the MetaboLights repository. Processed data generated during this study are included in this published article (and its Supplementary Information files).

## Electronic supplementary material

Supplementary Data 1

Supplementary Dataset 1

## References

[1] Ifa DR, Gumaelius LM, Eberlin LS, Manicke NE, Cooks RG (2007). Forensic analysis of inks by imaging desorption electrospray ionization (DESI) mass spectrometry. Analyst.

[2] Jones RW, Cody RB, McClelland JF (2006). Differentiating writing inks using direct analysis in real time mass spectrometry. Journal of forensic sciences.

[3] Weyermann C, Marquis R, Mazzella W, Spengler B (2007). Differentiation of blue ballpoint pen inks by laser desorption ionization mass spectrometry and high-performance thin-layer chromatography. J. Forensic Sci..

[4] Dunn JD, Allison J (2007). The detection of multiply charged dyes using matrix-assisted laser desorption/ionization mass spectrometry for the forensic examination of pen ink dyes directly from paper. J. Forensic Sci..

[5] Sotheby’s. (n.d.). The library of an English bibliophile, Part 1; Poems, chiefly in the scottish dialect, Kilmarnock. Retrieved August 17, from http://www.sothebys.com/en/auctions/ecatalogue/lot.23.html/2010/the-library-of-anenglish-bibliophile-part-1-l10411 (2017).

[6] CABI. Robert Burns’ Ode to tourism. Retrieved August 17, from (2017) https://www.cabi.org/leisuretourism/news/5201 (2003).

[7] Fergus, D. “Antique Smith” the Affable Forger: Textualities. Retrieved August 17, 2017, from http://textualities.net/david-fergus/antique-smith-the-affable-forger (2009).

[8] Newspaper cuttings of the Alexander ‘Antique’ Smith trial (held in the Robert Burns Birthplace Museum, Alloway) (1892).

[9] Ferguson, J. D. L. Antique Smith and His Forgeries. *The Colophon*, Ch. 13, (Pynson Printers, 1933).

[10] Hahn O, Malzer W, Kanngiesser B, Beckhoff B (2004). Characterization of iron-gall inks in historical manuscripts and music compositions using x-ray fluorescence spectrometry. X-Ray Spectrom.

[11] Klockenkämper R, Von Bohlen A, Moens L (2000). Analysis of pigments and inks on oil paintings and historial manuscripts using total reflection x-ray fluorescence spectrometry. X-Ray Spectrom.

[12] Hahn O, Kanngießer B, Malzer W (2005). X-ray Fluorescence Analysis of Iron Gall Inks, Pencils and Coloured Crayons. Stud. Conserv..

[13] Lee AS, Mahon PJ, Creagh DC (2006). Raman analysis of iron gall inks on parchment. Vib. Spectrosc..

[14] Kiefer W, Mazzolini aP, Stoddart PR (2007). Recent Advances in linear and nonlinear Raman spectroscopy I. J. Raman Spectrosc..

[15] Burgio L, Clark RJH, Hark RR (2010). Raman microscopy and x-ray fluorescence analysis of pigments on medieval and Renaissance Italian manuscript cuttings. Proc. Natl. Acad. Sci. USA.

[16] Ifa DR, Gumaelius LM, Eberlin LS, Manicke NE, Cooks RG (2007). Forensic analysis of inks by imaging desorption electrospray ionization (DESI) mass spectrometry. Analyst.

[17] Williams MR (2009). Analysis of black writing ink by electrospray ionization mass spectrometry. Forensic Sci. Int..

[18] Jones RW, Cody RB, McClelland JF (2006). Differentiating writing inks using direct analysis in real time massspectrometry. J. Forensic Sci..

[19] Ifa DR, Jackson AU, Paglia G, Cooks RG (2009). Forensic applications of ambient ionization mass spectrometry. Anal. Bioanal. Chem..

[20] Rogers, S. & Girolami, M. A First Course in Machine Learning (1st ed.). Chapman & Hall/CRC (2011).

[21] Bodzon-Kulakowska A, Drabik A, Ner J, Kotlinska JH, Suder P (2014). Desorption electrospray ionisation (DESI) for beginners–how to adjust settings for tissue imaging. Rapid Communications in Mass Spectrometry: RCM.

[22] Benjamini, Y. & Hochberg, Y. “Controlling the false discovery rate: a practical and powerful approach to multiple testing” (PDF). *Journal of the Royal Statistical Society, Series B*. **57**(1): 289–300 MR 1325392 (1995).

