# Supplementary material for: Minimally-destructive atmospheric ionisation mass spectrometry authenticates authorship of historical manuscripts
Source: Sci Rep. 2018 Jul 26;8:10944. doi: 10.1038/s41598-018-28810-2 (PMC6062563; doi:10.1038/s41598-018-28810-2)
Supplement: Supplementary file 1 — Supplementary Data 1 [file 41598_2018_28810_MOESM1_ESM.docx]

# Minimally-destructive atmospheric ionisation mass spectrometry authenticates authorship of historical manuscripts

James Newton^1,2^, Gordon Ramage^3^, Nikolaj Gadegaard^4^, William Zachs^5^, Simon Rogers^6^, Michael P. Barrett^1,2^, Gerard Carruthers^7^, Karl Burgess^1,2*^

^1^ Glasgow Polyomics, University of Glasgow

^2^ Wellcome Centre for Molecular Parasitology, Institute of Infection, Immunity and Inflammation, University of Glasgow

^3^ Dental School, University of Glasgow

^4^ School of Engineering, University of Glasgow

^5^ School of Literature, Language and Culture, University of Edinburgh

^6^ School of Computing Science, University of Glasgow

^7^ School of Critical Studies, University of Glasgow

# Supplementary Data 1

List of manuscripts

A number of authentic manuscripts were obtained for analysis from a private collection, these include 9 by Alexander Howland Smith (aka antique Smith), 7 of which are written in the hand of R. Burns and 3 original manuscripts from Robert Burns.

| **Sample ID** | **Author** | **Description** |
| --- | --- | --- |
| **MSS1** | A. Smith | The Holy Fair in the hand of R. Burns, also signed by J. Hogg |
| **MSS2** | A. Smith | Letter 1 in the hand of R. Burns |
| **MSS3** | A. Smith | Letter 2 in the hand of R. Burns |
| **MSS4** | A. Smith | Dainty dive poem in the hand of R. Burns |
| **MSS5** | A. Smith | Dainty dive poem 2 in the hand of R. Burns |
| **MSS6** | A. Smith | The first psalm in the hand of R. Burns |
| **MSS10** | R. Burns | A note written by R. Burns |
| **MSS11** | R. Burns | A letter written by R. Burns |
| **MSS13** | A Smith | Letter 3 in the hand of R. Burns |
| **MSS14** | R. Burns | The Five Carlins poem |

**Supplementary Table S1:** Authentic manuscripts descriptions along with their assigned sample ID.

Supplementary Figure S1


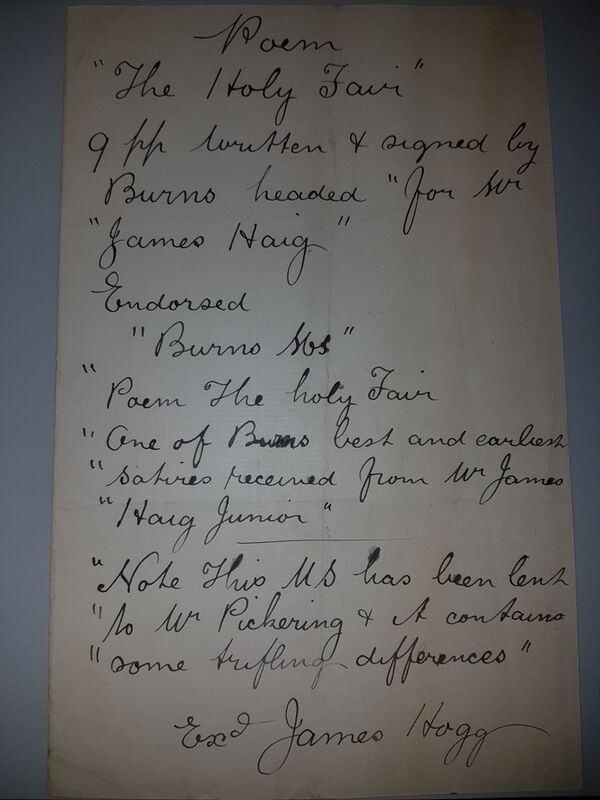

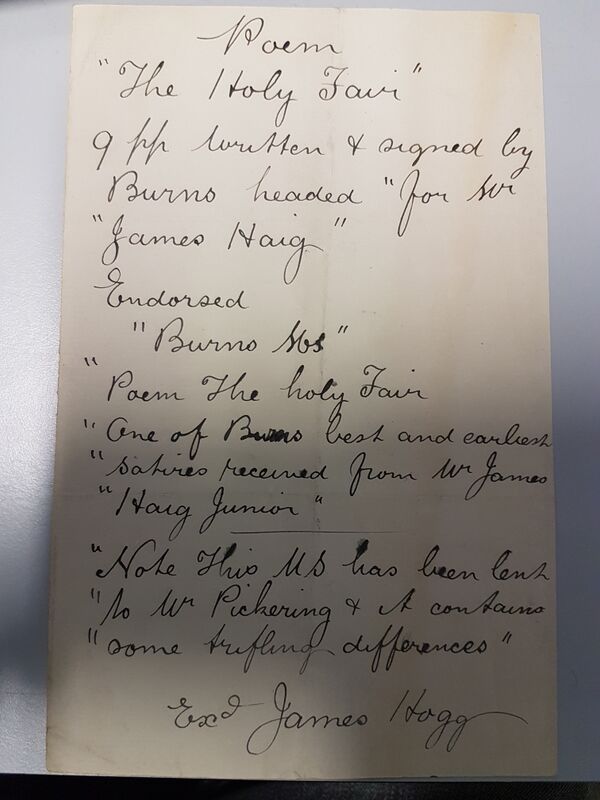


Demonstration that the technique is minimally destructive: red arrows indicate sampling locations. Left: Image of manuscript MSS1 before sampling. Right: Image of manuscript MSS1 after sampling

Supplementary Figure S2

The spectra below demonstrate four unique ions that can be used to identify A. Smith and R. Burns manuscripts.


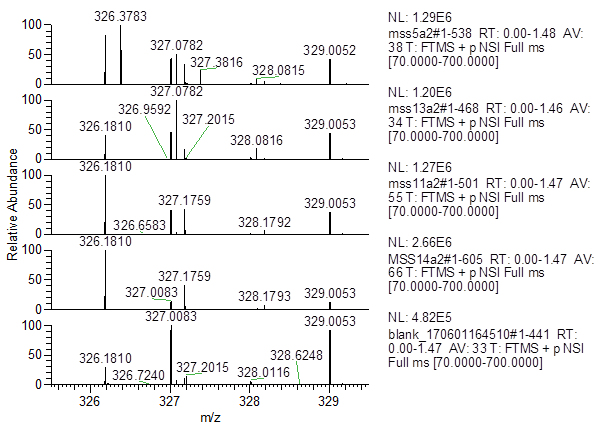


**MSS5**

**MSS13**

**MSS11**

**MSS14**

**Blank**

Showing the unique peak at *m/z* 327.0782 for a selection of manuscripts sampled from an area of ink, from the top to bottom A. Smith; MSS5 & MSS13, R. Burns; MSS11 & MSS14 and solvent blank.

Supplementary Figure S3


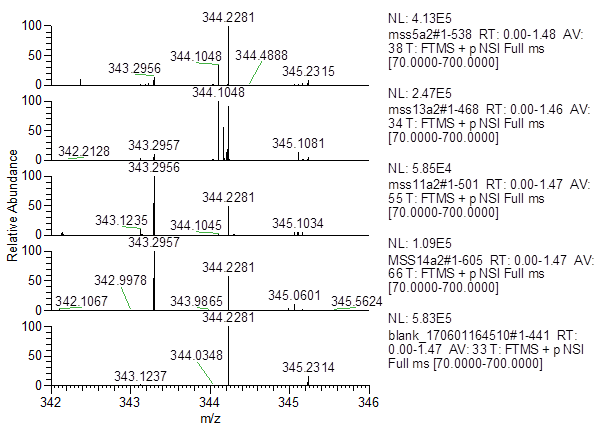


**MSS5**

**MSS13**

**MSS11**

**MSS14**

**Blank**

Showing the unique peak at *m/z* 344.1048 for a selection of manuscripts sampled from an area of ink, from the top to bottom A. Smith; MSS5 & MSS13, R. Burns; MSS11 & MSS14 and solvent blank.

Supplementary Figure S4


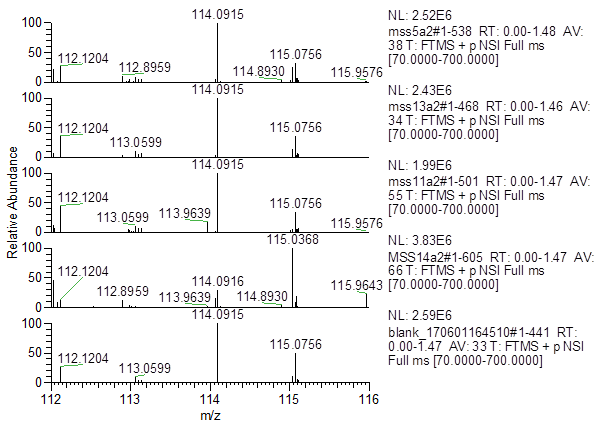


**MSS5**

**MSS13**

**MSS11**

**MSS14**

**Blank**

Showing the unique peak at *m/z* 113.9639 for a selection of manuscripts sampled from an area of ink, from the top to bottom A. Smith; MSS5 & MSS13, R. Burns; MSS11 & MSS14 and solvent blank.

Supplementary Figure S5

**MSS5**

**MSS13**

**MSS11**

**MSS14**

**Blank**

Showing the unique peak at *m/z* 230.9904 for a selection of manuscripts sampled from an area of ink, from the top to bottom A. Smith; MSS5 & MSS13, R. Burns; MSS11 & MSS14 and solvent blank.

Supplementary Figure S6

The selection of manuscripts selected demonstrate that a unique peak at *m/z* 273.0289 are only present in A. Smith manuscripts and not R. Burns or the solvent blanks, the unique peak was present in all A. Smith manuscripts and no R. Burn manuscripts or solvent blanks.

**MSS5**

**MSS13**

**MSS11**

**MSS14**

**Blank**

Demonstrating the absence of the unique peak at *m/z* 273.0289 for R. Burns for MSS11 & MSS14, and presence of the unique peak at *m/z* 273.0289 for A. Smith for MSS5 & MSS13. The final row demonstrates the absence of the feature in a solvent blank.
